# Methylation of free-floating deoxyribonucleic acid fragments in the bronchoalveolar lavage fluid of dogs with chronic bronchitis exposed to environmental tobacco smoke

**DOI:** 10.1186/s13620-015-0035-4

**Published:** 2015-04-29

**Authors:** Yoshiki Yamaya, Hiroshi Sugiya, Toshihiro Watari

**Affiliations:** Laboratory of Comprehensive Veterinary Clinical Studies, Department of Veterinary Medicine, College of Bioresource Sciences, Nihon University, Kameino 1866, Fujisawa, Kanagawa Japan; Laboratory of Veterinary Biochemistry, Department of Veterinary Medicine, College of Bioresource Sciences, Nihon University, Kameino 1866, Fujisawa, Kanagawa Japan

**Keywords:** Chronic bronchitis, DNA methylation, Dog, Second-hand smoke

## Abstract

**Background:**

The etiology of canine chronic bronchitis (CB) is not completely understood, although exposure to environmental tobacco smoke (ETS) affects the airway inflammatory responses in some dogs with CB. The mechanism by which this occurs is unknown.

**Findings:**

We investigated the concentrations and methylation rates of free-floating DNA fragments in bronchoalveolar lavage fluid (BALF) from dogs with chronic bronchitis. Based on serum cotinine levels, dogs with CB were divided into 2 groups: dogs that either had or had not been exposed to ETS. Our results demonstrated that the total nucleated cell and macrophage numbers increased in BALF of ETS-exposed dogs with CB. There were no significant differences in DNA concentrations and methylation rates in BALF between the 2 groups. However, 3 out of 8 dogs exposed to ETS had high DNA methylation rates in their BALF samples.

**Conclusion:**

Our results suggest that ETS exposure leads to epigenetic modifications of cellular components in BALF in dogs diagnosed with CB.

## Findings

Deoxyribonucleic acid (DNA) methylation is an epigenetic modification that mainly occurs in CpG dinucleotides in the CpG-rich islands located within gene promoter regions. Methylation of CpG islands in promoter regions is generally associated with gene silencing. Another epigenetic modification, DNA hypomethylation, is associated with gene transcription [1]. The level of epigenetic DNA methylation is considered to be an important factor in the pathogenesis of various human diseases, including pulmonary diseases such as lung cancer and chronic obstructive pulmonary disease (COPD), a pulmonary disorder associated with exposure to cigarette smoke [2-4]. In humans, tobacco smoke exposure causes changes, such as DNA methylation that are reversible and DNA mutations that are irreversible, to the epigenetic status [5-10].

In dogs, environmental tobacco smoke exposure (ETS) plays a role in various smoking-related respiratory conditions [11,12]. When mice were exposed to ETS, the overall response was primarily global DNA hypomethylation in the lung [13]. This finding is most consistent with our current understanding of ETS-induced carcinogenesis. In contrast, in cultured human airway epithelial cells exposed to ETS, the induction of locoregional DNA hypermethylation in the CpG-rich gene promoter region was observed [14].

The etiology of canine chronic bronchitis (CB) is not completely understood, although ETS exposure has additional effects on the airway inflammatory responses in some dogs with CB [12]. We examined the effect of ETS exposure on airway inflammatory responses. We measured the concentrations and methylation rates of free-floating DNA fragments in the bronchoalveolar lavage fluid (BALF) from dogs diagnosed with CB who had or had not been exposed to ETS. We compared the changes in these parameters between the two groups.

## Methods

Nineteen dogs (4–12 years; 11 males and 8 females) diagnosed with CB were included in this study. Between April 2005 and March 2010, these dogs had been referred to the Animal Medical Center of Nihon University for diagnoses and consultations for long-term therapy for their clinical signs. The breeds included Miniature Dachshund (n = 12), Shih Tzu (n = 2), and 1 each of Pug, Maltese, Papillon, Shetland sheepdog, and mixed breed. CB was diagnosed based on normal ranges of blood test results, thickened bronchial walls on chest X-ray and computed tomography, mucosal secretions, and patterned indented surface in bronchoscopy. Total nucleated cell counts were determined and the results were used, provided the dogs did not present with other nasal, throat, cardiac, infectious and neoplastic diseases. Either an elevated total nucleated cell count (> = 400 cells/μL) with macrophage dominance (> = 50%) or normal total nucleated cell count (<400 cells/μL) with neutrophil dominance (> = 15%) in the BALF samples was used as one of the parameters for the inclusion criteria. All examinations were performed after informed consent of the dogs’ owners under the guideline for the care and use of laboratory animals by The College of Bioresource Sciences, Nihon University.

Blood samples were collected from jugular veins as in routine clinical examinations. Serum was isolated by centrifugation and stored at −20°C until used for cotinine measurements. Serum cotinine was determined using a Cotinine Passive Smoking ELISA Kit (Cosmic Corporation, Tokyo, Japan). BALF samples from the dogs were obtained by gentle aspiration through a biopsy channel of a bronchoscope after infusing sterile saline (0.9% NaCl) solution (40 mL, divided into 2 aliquots) under general anesthesia with isoflurane. BALF sample supernatants were isolated by centrifugation (200 *× g* for 10 min) and immediately stored at −20°C until DNA extraction. Free-floating DNA in 200 μL of BALF supernatant was concentrated to 20 μL using a DNA Extractor Kit (Wako, Japan). Concentrations of the extracted DNA in sera and BALF samples were determined using a Nanodrop Spectrophotometer (NanoDrop Technologies, USA) and corrected by urea concentrations (QuantiChrom^TM^ Urea Assay Kit, BioAssay Systems, Hayward, CA, USA).

DNA methylation rates were determined with a Methylamp Global DNA Methylation Quantification Ultra Kit (Epigentek, USA) according to the manufacturer’s instructions. The capture antibody in this kit binds to 5-methylcytosine. The total DNA methylation level was determined as a percentage of the total DNA in a given sample. This technique measured the total DNA fragments in the samples but not specific sites, patterns, or methylation types. Samples were run in duplicate and points for the standard curve were run in triplicate.

Medians with the range of minimum and maximum values were used to express results. Group results were compared by the Mann–Whitney rank sum test for non-normally distributed variables. P values < 0.05 were considered significant. All statistical analyses were performed using SigmaPlot for Windows, Version12.0 (Systat Software, Inc., San Jose, CA, USA).

## Results

Based on serum cotinine levels, 19 dogs with CB were divided into 2 groups: dogs who either had or had not been exposed to ETS. Eleven dogs (4–12 years) not exposed to ETS and 8 dogs (6–11 years) exposed to ETS were analyzed. The results from their BALF samples are shown in Table 1. Total nucleated cell counts and the numbers of macrophages in BALF from dogs exposed to ETS were significantly higher than those of the dogs not exposed to ETS. There were no significant differences in DNA concentration (P = 0.386), corrected DNA concentration (P = 0.756), and DNA methylation (P = 0.076) in the BALF samples. The rate of DNA methylation in 3 of the 8 dogs exposed to ETS was higher than the range of that rate in dogs not exposed to ETS (Table 1).

**Table 1:** **Results from bronchoalveolar lavage from dogs with chronic bronchitis that had or had not been to environmental tobacco smoke**

	**Unexposed dogs**	**Exposed dogs**
Number	11	8
Males:Females	8:3	3:5
Age(y)	7.2 ± 2.5	8.6 ± 2.3
Serum cotinine levels (ng/mL)	0 (0–0)	0.41 (0.03–1.42)*
Cells in BALF		
Total nucleated cell counts (cells/μL)	124 (57–231)	600 (100–3175)*
Macrophages (%),	56 (8–79)	73 (50–92)
(cells/μL)	56 (9–148)	438 (50–2921)*
Neutrophils (%),	21 (2–82)	12 (0–27)
(cells/μL)	30 (3–178)	26 (0–345)
Lymphocytes (%),	19 (3–54)	7 (0–36)
(cells/μL)	24 (2–78)	35 (0–870)
Eosinophils (%),	0 (0–4)	0 (0–3)
(cells/μL)	0 (0–4)	0 (0–20)
Total DNA concentrations (ng/μL)	7.0 (3.3–20.2)	5.7 (2.4–29.4)
Corrected DNA concentrations (ng/μL)	413 (158–1582)	478 (112–743)
Rates of DNA methylation (%)	11.4 (1.0–23.9)	17.0 (9.5–42.7)

*: significant differences between groups (*p* < 0.05).

## Discussion

It has been reported that acute tobacco smoke exposure induced alveolar macrophage apoptosis [15]. If the source of DNA fragments in BALF was alveolar macrophages, it would be expected that the DNA concentrations in BALF would be higher in dogs exposed to ETS than those not exposed to ETS. We found no differences in the DNA concentrations in the BALF samples in either group. In this study, the number of macrophages in dogs exposed to ETS was higher than those in dogs not exposed to ETS. This result is supported by the results from a previous report, where the phagocytic activity of macrophage was enhanced by ETS exposure in both healthy dogs and those with CB [16].

Damaged airway epithelial cells may also be a considerable source of free-floating DNA in BALF. Gene hypermethylation occurs in the bronchial epithelium of human smokers [5,10]. In human patients with COPD, gene methylation was enhanced in the sputum, which includes bronchial secretions with exfoliated cells from the lower respiratory tract [17]. In addition, 20% of atopic human patients develop asthma, which is usually exacerbated by environmental factors including air pollution or oxidative stress. DNA methylation in airway epithelial cells was reduced in atopic children but increased in asthmatic children with the histopathological response in airway epithelial cells [18]. The enhanced methylation of free-floating DNA fragments in BALF may be linked with the histopathological damage in airway epithelial cells caused by ETS exposure in dogs with CB [12].

The limitations of this study were the small sample size and an unknown source of free-floating DNA fragments in the BALF samples. In addition, there is no available information regarding normal methylation levels of free-floating DNA fragments in BALF from clinically healthy adult dogs. It is possible that DNA levels in BALF samples from healthy dogs may be undetectable or very low. Similar results were found in the airway samples from healthy human subjects [17].

## Conclusion

We conclude that ETS exposure may lead to epigenetic modifications of cellular components in BALF from dogs with CB. Further studies will be needed to determine the location of genes with aberrant methylation patterns and their therapeutic implications in dogs with CB that either have or have not been exposed to ETS. Theophyline and β2-adrenergic agonists may be therapeutic and beneficial for normalizing DNA methylation [2,19].

## Abbreviations

BALF: Bronchoalveolar lavage fluid
CB: Chronic bronchitis
DNA: Deoxyribonucleic acid
ETS: Environmental tobacco smoke
